# The source of the Nile

**DOI:** 10.1093/sleep/zsaf407

**Published:** 2025-12-22

**Authors:** Kingman P Strohl

**Affiliations:** University Hospitals Cleveland Medical Center, Department of Physiology and Biophysics, Case Western Reserve University, Cleveland, OH, United States

Herodotus in the 5th century BCE called the Nile River Delta the “gift,” describing its tributaries, wetlands, and populated islands, and told of its glories, in particular the library at Alexandria where all knowledge was recorded. Much later, explorers and geologists mapped the river to the Blue Nile and the White Nile. The Nile River itself travels about 4132 miles depositing its fruits (water, sediment, commerce, etc.), creating the Delta. Our current knowledge about obstructive sleep apnea (OSA) can be described as the Delta; here this essay considers its source.

**Figure 1:**
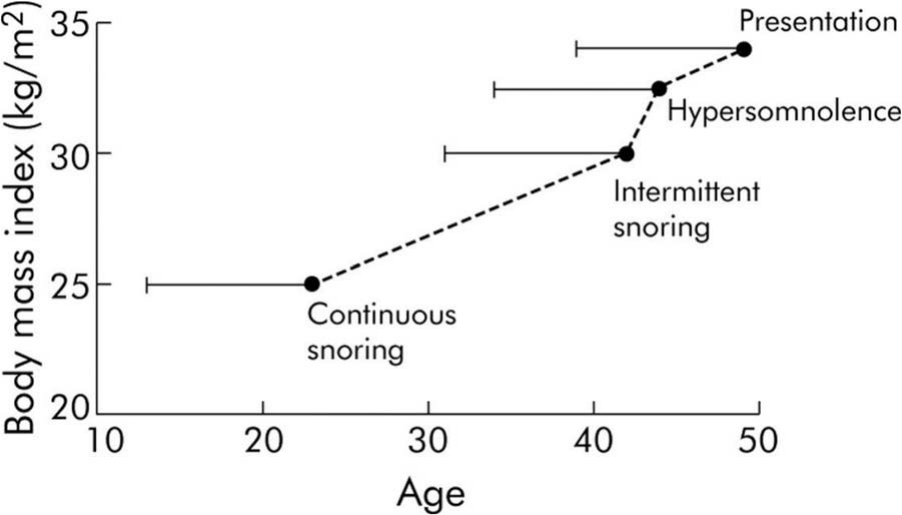
This figure is one published in a chapter (Lugaresi et al., 1990) and is the first model describing for adult OSA a timeline for development based upon the recalled snoring and BMI, and proceeding to symptom development, in this case hypersomnolence and then clinical presentation (published by permission of Raven Press).

In the great library of OSA, four important papers were recorded in the early years. Remmers et al. [1] showed evidence from the genioglossus muscle electromyogram during sleep showing fluctuating brainstem motor control causing obstruction in that tube that evolutionarily and physiologically is the connection to the gas exchanging units. The Stanford crowd [2] described the brainstem in sleep as coordinating muscle activation to keep the airway open, not by individual muscles, but mechanically by proposing that the stylopharyngeus opened airway by pulling on the constrictor muscles opening the pharynx, the “tent hypothesis.” Next, theme of feedback control explained the respiratory dysrhythmias of sleep [3] through illustrations of acute and/or chronic deficiencies in sensation (afferent feedback), coordination of neural processing and output (brainstem elements), and the controlled system (nerves, muscles, and structural features including the cortical arousals). Then key to this article, in 1990 [4] was described a two decade experience with heavy snorer’s disease from local towns and suggested that continuous snoring in one’s 20s developed into discontinuous snoring in one’s 40s before symptoms by age 50 and then a diagnostic presentation a few years later (Figure 1). We know now that BMI is a major but not monolithic feature in adult OSA presentations, but this was the first description of a “latent period” in the process upstream to disease.

Our OSA library describes a functional problem—an inability to keep the airway open enough as to not interrupt sleep too much. Unlike locomotion which is a function learned and mastered after the first year through trial and error, the resiliency and adaptivity in the upper airway starts with birth breathing, sucking, and swallowing, and adjusts to state, position, and changes to the working upper airway anatomy [5]. The system evolved rather well in all vertebrates. In infants, baring congenital deformities, detection of problems and cooperation among clinicians and basic scientists resulted in successful primary prevention with “back to sleep” public service announcements, and reductions sudden unexplained death. In adult humans, the function works excellently with healthy breathing through the upper airway during sleep, the inverse of OSA, estimated at 90%–95% of children [6] and ~75% of adults [7]. So how do people “catch” OSA.

In considering this latent period, one can list those risk factors/co-morbidities which might predispose to upper airway sleep instability. Obesity, as it develops over time has both structural and humoral elements that affect brainstem functions [8]. Hypertension is another common feature of adult OSA, but hypertension alone without OSA has a “latent phase” with longstanding endothelial pathophysiology; and the brainstem like other organs benefits from a healthy endothelium. In rodent models of hypertension, there are changes in brainstem regulation of autonomic outputs and carotid afferent activity affecting it [9]. Consider diabetes, there are early neuropathic features and changes brainstem circuits present in rodent models of type 2 diabetes [10]. Likewise, aging, gender, hormones, and menopause affect intrinsic brainstem functions—frequency, tidal volume, and patterning in healthy individuals. Often these are studied physiologically, isolating features relevant to sleep disordered breathing. Moreover, the literature on “OSA models” currently emphasizes exposure to OSA consequences (intermittent hypoxia or to arousals) on brain and cardiovascular pathophysiology [11]. In these models, assessments of the brainstem circuitry have a focus on breath generation, neural coordination, and/or interactions with swallowing or reflexes, without much on mechanical effectors or circuit instability during sleep.

**Figure 2:**
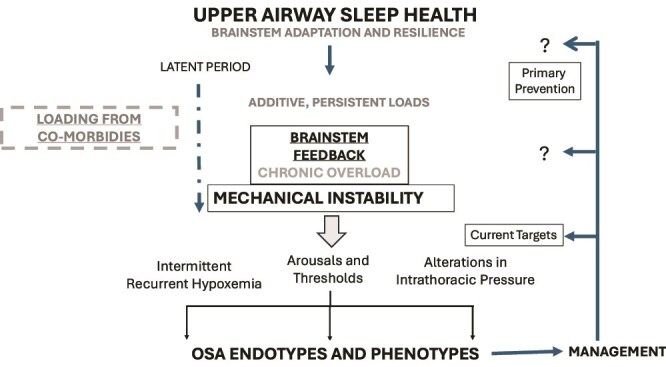
A healthy airway can handle the need for ventilation during sleep as well as wakefulness using a feedback control system with the brainstem as the controller and adapts to growth and development. This acutely resilient system often works well, but additional stressors can accumulate over time and the brainstem feedback control is unable to compensate. The proposal is that in this early phase functions become inherently unstable through the agency of chronic anatomic and metabolic factors, including co-morbid loads of other diseases or behavioral factors. These confound the controller possibly by inflammation/oxidation, and remodeling, to start one on the path to sleep disordered breathing. As the intensity of events increases, so do the consequences which can also alter the feedback system. Clinical presentations traits, shaped by genes and the consequences of instability, are the personalized traits in the presentation. We treat now the upper airway instability but should look to a more upstream opportunity for primary prevention.

Figure 2 is a sketch of the processes from the origins of successful function and the elements of a “latent period” between the starting of pathophysiology and the expression of disease. Setting aside fatal or handicapping birth defects, “upper airway sleep health” is the set point for most of us at birth. The upper airway can adapt its function for sleep state, position, sucking, and swallowing, which evolves through growth, development, and aging. The controller is resilient in the context of temporary challenges from nasal obstruction by viral illnesses, adenotonsillar hypertrophy, and alcohol. The idea is that over time stressors could build up and the brainstem gradually loses its feedback ability, not suddenly, but progressively, to move to something, like as proposed chronic snoring. Then one or more conditions may further stress its ability, a failure to maintain stability until arousals occur, intermittent changes in gas exchange, and alterations in intrathoracic pressure, all affecting vascular pressures. Some of these events load the control system by changing afferent input or intrinsic gain. In short, both early and late brainstem responses could exacerbate controller functions. In this scenario, the comorbid risks play a role. Those with reasonably healthy controllers flow through the Delta tributaries concluding the lifecycle.

The manifestations of a dysfunctional upper airway (apneas, hypopneas, RERAs, etc.) ebb into the different marshes; some would be called phenotypes (presenting consequences of events) and islands, endotypes (causal pathways for recurrent apnea) [12]. Genetic polymorphisms may play a role in causing “personalized” consequences to the root cause—chronic instability. Gaps in our knowledge include the identification of the upstream events.

Wear and tear that accumulates in this latent period from genetic, environmental, or psychological sources adds up non-linearly [13]. Tailoring this concept to OSA, the presenting co-morbidity—obesity, diabetes, hypertension, cardiovascular disease—are endogenous, and smoking/alcohol are exogenous stressors. Socioeconomic factors are psychological and food availability, habits, etc. add up. Sleep deprivation whether due to anxiety, depression, or a hectic lifestyle, can over time impair function and contribute to expression and progression [14] and can contribute to expression of disordered breathing [15].

Even genetic risk, like paired-like homeobox 2b (PHOX2B), which may present as congential central hypoventilation syndrome (CCHS) in an infant, can also present after a “latent period,” in the second decade or in adult close-relatives, when the tandem repeat is not too long [16]. Like hypertension, adult OSA develops as a complex disease where no one gene or factor produces the condition of high blood pressure [17]. In adult presentations, there may be many polymorphisms lining up with 3%–5% effect or a combination of genes and non-genetic factors over a timeline of months to years which add up to push the system into instability and produce disability. Sleep mechanisms may operate through arousal thresholds leaving more or less time to adjust muscle actions to a mechanical load, the latter leading to higher loop gain [18]. A loss of sensory feedback [19] or insulin resistance [20] accumulate. Anxiety and insomnia may contribute to chronic instability or the expression of symptoms or vascular consequences [21]. These are broad issues at present, with the details in this multidimensional “latent period” needing actionable definitions.

To make our way back to the metaphor, we have traced the source of the Nile to its Blue Nile (genetics) and White Nile (environment) ancestry that formed upper airway function, and navigated down the Nile bumping into challenges of growth and development, aging, gender, and forces—socioeconomics, behavior, and illness—which both directly and indirectly affect the ability to maintain flow. As adaptability and resiliency degrades, one or more factors can produce chronic feedback instability during sleep producing the common and multifaceted OSA presentations. These forces are recognized every clinic day at the mouth of the Nile, starting with age, sex, comorbidity, drugs, alcohol, smoking, etc., and counting the expressed symptoms and pathways. So, when one reads a sleep study of a 49-year-old and finds an AHI ~10/h or more, do not assume that he/she “caught it” last year. Consider what upstream contributions are present, and when/how the start might be identified 5, 10, or 15 years earlier. Multidisciplinary investigations are needed to bend the curve of the expected future torrent of OSA toward early detection and prevention.
